# *De novo STXBP1* Mutations in Two Patients With Developmental Delay With or Without Epileptic Seizures

**DOI:** 10.3389/fneur.2021.804078

**Published:** 2021-12-24

**Authors:** Ping Yang, Robert Broadbent, Chitra Prasad, Simon Levin, Sharan Goobie, Joan H. Knoll, Asuri N. Prasad

**Affiliations:** ^1^Department of Pathology and Laboratory Medicine, Western University, London, ON, Canada; ^2^London Health Sciences Centre, London, ON, Canada; ^3^Department of Paediatrics, London Health Sciences Centre, Western University, London, ON, Canada; ^4^Maritime Medical Genetic Service, Department of Paediatrics, Izaak Walton Killam Health Centre, Halifax, NS, Canada

**Keywords:** *STXBP1*, haploinsufficiency, dominant negative, nonsense mutation, RNA expression

## Abstract

**Objectives:** Mutations in the STXBP1 gene have been associated with epileptic encephalopathy. Previous studies from *in vitro* neuroblastoma 2A cells showed that haploinsufficiency of *STXBP1* is the mechanism for epileptic encephalopathy. In this *ex vivo* study, *STXPB1* DNA mutations and RNA expression were assessed from two patients to help understand the impact of *STXBP1* mutations on the disease etiology and mechanism.

**Methods:** Microarray analysis and DNA sequencing were performed on two children with development delay, one with and one without infantile spasms. Different pathogenic mutations of *STXBP1* were identified in the patients and RNA expression of *STXPB1* was then performed by RT-Q-PCR on RNA extracted from blood samples of each patient.

**Results:** Pathogenic deletion [of exons 13–20 and 3′ downstream of *STXBP1*] and nonsense mutation [c.1663G>T (p.Glu555X) in exon 18 of *STXBP1*] were detected from the two patients, respectively. RNA analysis showed that 1) the deletion mediated RNA decay, and that 2) no RNA decay was identified for the nonsense mutation at codon 555 which predicts a truncated STXBP1 protein.

**Significance:** Our RNA expression analyses from the patient blood samples are the first *ex vivo* studies to support that both haploinsufficiency and truncation of STXBP1 protein (either dominant negative or haploinsufficiency) are causative mechanisms for epileptic encephalopathies, intellectual disability and developmental delay. The RNA assay also suggests that escape from nonsense-mediated RNA decay is possible when the nonsense mutation resides <50 nucleotides upstream of the last coding exon-exon junction even in the presence of additional non-coding exons that are 3′ downstream of the last coding exon.

## Introduction

Missense, nonsense, frame shift mutations and deletions of all or part of the *STXBP1* gene have been reported in the literature in more than 280 patients affected with epileptic encephalopathy patients with early infantile onset-4 (OMIM # 612164). All patients with germline pathogenic variants and deletions of *STXBP1* have global developmental delay, intellectual disability and cognitive dysfunction. The majority of affected patients present with seizures (1). Mutations or deletions of *STXBP1* have been reported primarily in patients affected with Ohtahara syndrome (2, 3), West syndrome (4), and less frequently in patients affected with early myoclonic epileptic encephalopathy (5, 6), Dravet syndrome (7), Lennox-Gaustaut syndrome (8), Angelman/Pitt Hopkins-like syndrome phenotype (9, 10), and atypical Rett/Rett-like phenotypes (11). Ohtahara syndrome is characterized by neonatal onset severe seizures, tonic spasms, burst suppression pattern on EEG, intellectual disability and developmental delay (2). West syndrome is defined by infantile spasm onset between 3 and 12 months of age with atypical hypsarrhythmia (4). Recently, mutations or deletions of *STXBP1* have been reported in 14 patients with intellectual disability without epileptic seizures (12, 13). Somatic mutation of *STXBP1* was also identified in a patient with focal cortical dysplasia (14).

*STXBP1* encodes syntaxin binding protein 1 which is highly expressed in brain and plays a role in neurotransmitter release as part of the synaptic fusion machinery. There are two isoforms of the *STXBP1* gene. One isoform (isoform a) contains 20 exons (NM_003165) encoding 603 amino acids (P61764-2) and another isoform (isoform b) has 19 exons (NM_001032221) encoding 594 amino acids (P61764-1), respectively. The difference between the two isoforms at the amino acid sequence level is from positions 576 to 594 or to the C-terminal end (3). Mutations in *STXBP1* can be detected by sequencing analysis for 83% of cases, by targeted deletion/duplication analysis for 5% of cases and by microarray analysis for 12% of cases (7). Haploinsufficiency of *STXBP1* has been proposed as the mechanism for the epileptic encephalopathies based on expression experiments of mutant STXBP1 proteins in cultured neuroblastoma 2A cells (3). Recently, a dominant negative mechanism has been suggested from missense mutant studies (15).

In this study, we report genomic and RNA findings on two unrelated children with different *STXBP1* alterations. The results are presented for each proband in the Genetic Anomalies and *STXBP1* Expression sections. Our findings from patient blood samples are the first *ex vivo* assay to support that both haploinsufficiency and truncation of STXBP1 protein (either dominant negative or haploinsufficiency) are causative mechanisms for epileptic encephalopathies, intellectual disability and developmental delay.

## Materials and Methods

### Patients

Proband 1: A 2 year old male child was referred for microarray testing due to clinical findings of developmental delay, intellectual disability and hypotonia. An intragenic deletion of the *STXBP1* gene was detected by microarray analysis. Parental blood samples were obtained for follow-up studies to determine if the deletion was inherited or *de novo*.

Proband 2: A 6 year old child, with a history of infantile spasms, was tested with a 51-gene infantile epilepsy panel (GeneDx, Gaithersburg, Maryland, USA). A nonsense mutation, c.163G>T (p.Glu555X), was identified in exon 18 of *STXBP1*.

### DNA and RNA Extractions

DNA was extracted from peripheral blood cells from the probands, their parents, and five normal control males using the MagNA pure compact instrument and MagNA Pure LC DNA isolation kit 1 (Roche Diagnostics, Laval, Quebec, Canada). RNA was extracted from blood samples from the probands, and two normal male controls of similar age (1 and 6 years) using the RNeasy mini kit (QIAamp^®^ RNA Blood Mini, Qiagen, Hilden, Germany).

### Microarray Analysis

Approximately 200 ng of genomic DNA was used for the ThermoFisher High Resolution CytoScan HD Array studies according to the manufacturer's protocol. Genomic copy number variants (CNVs) and absence of heterozygosity (AOH) were identified using ChAS software from Affymetrix/ThermoFisher Scientific (Waltham, MA, USA).

### Relative Quantitative-PCR (Q-PCR) Analysis

**Figure 1:**
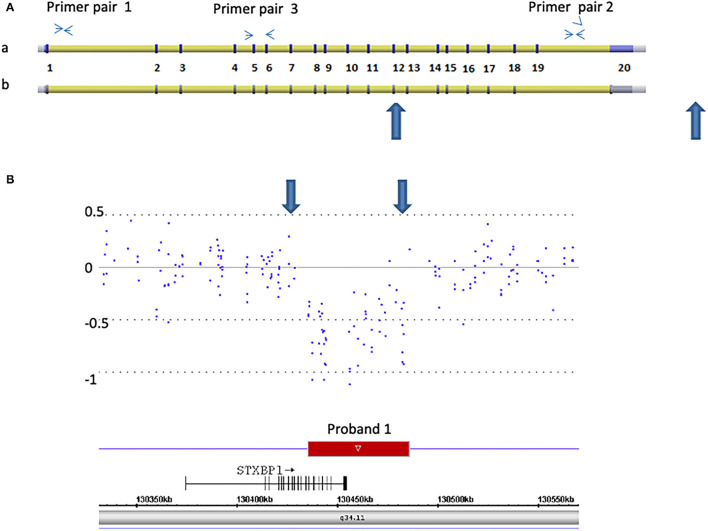
Detection of a 50 kb deletion of *STXBP1*, exons 13 through 20 and 3′ downstream genomic region in proband 1. **(A)** Schematic representation of two isoforms of *STXBP1* (a: NM_003165. b: NM_001032221) (not scaled). The exons (numbered 1–20) and introns are shown. Primer pairs 1 and 2 are located at introns 1 and 19, respectively. They were used for Q-PCR analysis. Primer pair 3 is located at exons 5 and 6 and was used for RT-Q-PCR analysis. **(B)** Microarray scan of ~250 kb region encompassing the *STXBP1* region. CytoScan HD array probes over the region are represented as dots. The numbers on the Y-axis represent Log2 values. The rectangle represents the region deleted. The *STXBP1* gene relative to the deletion (small arrow indicates the orientation from 5′ to 3′ of the gene), genomic location [hg19], and chromosome 9 band is indicated. Large blue arrows demarcate the region deleted in both **(A,B)**.

To confirm the small deletion and determine the inheritance of the deletion, Q-PCR studies were performed on the genomic DNA extracted from proband 1, parents and five normal controls. Two pairs of primers designed from within the hemizygously deleted region and undeleted region were used, respectively for the Q-PCR studies. Primer pair 1 is located in a non-deleted region in *STXBP1* intron 1: 5′ GACATTTGCAAAACGGCATC 3′-F and 5′ TGTTGGTGATGAGAAAGGTCA 3′-R (Figure 1A). Primer pair 2 is located within the deleted region in intron 19: 5′TTGCTTGTAACGAGGAAGCT 3′-F and 5′ TGAAGAGTGAACCATTGCCA 3′-R (Figure 1A). A *FOXP2* gene amplicon was used as a two-copy reference control for the relative Q-PCR calculation. It was amplified using primer pairs: 5′-TGCTAGAGGAGTGGGACAAGTA 3′-F and 5′ CAAAAGCCACAGCAATCCTT 3′-R (courtesy of TCAG, Hospital for Sick Children, Toronto, Canada). The relative Q-PCR amplification using 9 ng genomic DNA in a total 15 ul reaction volume was performed on the Roche LightCycler^®^480 real time PCR instrument using the LightCycler^®^ 480 high resolution melting master protocol. Briefly, the DNA was denatured at 95°C for 10 min, amplified at 95°C for 10 s, 60°C for 15 s, 72°C for 15 s for 45 cycles. The PCR products were melted at 95°C for 10 s, 65°C for 1 min, continuous at 95°C. The products were cooled to 37°C for 10 min. The LightCycler^®^480 software v1.5 using the ΔΔ Ct method determined the ratio of the amplified target sequence (*STXBP1*) to the amplified reference sequence (*FOXP2*) and normalized with the five pooled normal control samples (16–18). Each run contained triplicates for each sample and the mean number was obtained from the triplicates. Copy numbers were interpreted as loss (1n), normal (2n), and gain (3n) while the ΔΔ Ct ratios were 0.4–0.7, 0.8–1.2, and 1.3–1.7, respectively.

### Reverse Transcription-Q-PCR (RT-Q-PCR) Analysis

To determine if the deletion in proband 1 and the nonsense mutation in proband 2 mediate RNA decay, RT-Q-PCR studies were performed on RNA isolated from each proband and similar age- and gender- matched controls. Complementary DNA (cDNA) was obtained from reverse transcription (RT) of 2.5 ug total RNA using SuperScript III First-Strand Synthesis SuperMix (Invitrogen/ThermoFisher Scientific, Waltham, MA, USA). One pair of primers, outside of the *STXBP1* genomic deletion region, was designed on exons 5 and 6 (primer 3): 5′ TCTCATCAGTGACTTTAAGGACC 3′-F, 5′ AGTTTTGATGACTTTGGCTGCT 3′-R (Figure 1A) and was used for the RT-Q-PCR analysis.

PCR amplification was performed with the Applied Biosystems GeneAmpR PCR System 9700 (Applied Biosystems/ThermoFisher Scientific, Waltham, MA, USA) for 38 Cycles at 95°C denaturation for 10 s, annealing at 59°C for 15 s and extension at 72°C for 15 s. The PCR amplification was completed during the linear phase at cycle 38. The PCR products were separated on the TapeStation 2200 instrument with a High Sensitivity D1000 ScreenTape (Agilent Technologies, Waldbronn, Germany) as per the manufacturer's specifications for analysis of the RT-Q-PCR products. Data were obtained and images were captured using TapeStation Analysis Software (A.02.01) (Agilent Technologies, Waldbronn, Germany). The tests were repeated more than three times.

Consent forms were obtained from each family for this study. Research Ethics Boards (REB) approval was not required by our Institution based on the nature of the study (https://www.uwo.ca/research/_docs/ethics/hsreb_guidelines/Case_report_vs_research.pdf).

## Results

### Clinical Profiles

Proband 1: This male child presented with feeding difficulty and generalized hypotonia noted in the first few weeks after birth, delayed motor milestones (sitting at 12 months, walking with assistance at 24 months) as well as delays in both fine motor and language domains. At 2 years of age, he displayed behavioral stereotypies (hand flapping) and autistic traits (preoccupation with spinning objects). He has no history of seizures. His growth parameters were: height of 85 cm (10–25th%ile), weight of 11.85 kg (10%ile) and occipital frontal head circumference of 47 cm (3%ile) with a brachycephalic skull shape. Mild bilateral 5th finger clinodactyly was noted on physical examination, but no other significant dysmorphic features. Imaging studies of the brain showed increased periventricular T2 signal suggestive of delayed myelination. At age 3 years he was investigated for staring spells with an electroencephalogram (EEG) that showed mild background slowing, without any evidence of abnormal epileptiform activity. There were no sleep related changes documented on the available record. No other prior EEG recordings were available for review.

Proband 2: This 6 year old male presented to the neurogenetic clinic with a history of epileptic spasms with onset at 2 months of age, global developmental delay, poor visual attention (cortical visual impairment) and bilateral clubfeet with no major facial dysmorphism. His EEG findings at age 3 months confirmed the presence of discontinuous suppression burst pattern with multiple independent spike foci, features consistent with an epileptic encephalopathy. Subsequent follow up recordings done at regular intervals showed variable features with slow background rhythms, regional (occipital) expression of spike activity, with further enhancement and activation of spiking in sleep. By age 12 years the EEG recordings had begun to show features of generalized slow spike wave activity (2–2.5 Hz), paroxysmal fast (recruiting rhythms) in keeping with evolution of his epilepsy. Imaging studies showed non-specific features of generalized loss of gray matter volume. Extensive investigative work-up including biochemical studies for inborn errors of metabolism were negative. The patient was initially treated with Vigabatrin titrated to a maximum of 120 mg/kg/day, and further medication changes were dictated by appearance of refractoriness to therapy. Ophthalmological monitoring for retinal toxicity was continued. Valproic acid, and Levetiracetam were added sequentially and withdrawn due to a lack of benefit in terms of seizure control. Eventually, by age 4 years he was weaned off Vigabatrin. He did show a period of seizure remission for about 18 months around age 5 years, after which tonic spasms made a reappearance at age 7 years. At age 12 years, he continues to report nocturnal tonic seizures sometimes in clusters, and is currently maintained on Rufinamide 31 mg/kg/day in three divided doses daily and Clobazam 0.25 mg/kg at bedtime.

### Genomic Anomalies

**Figure 2:**
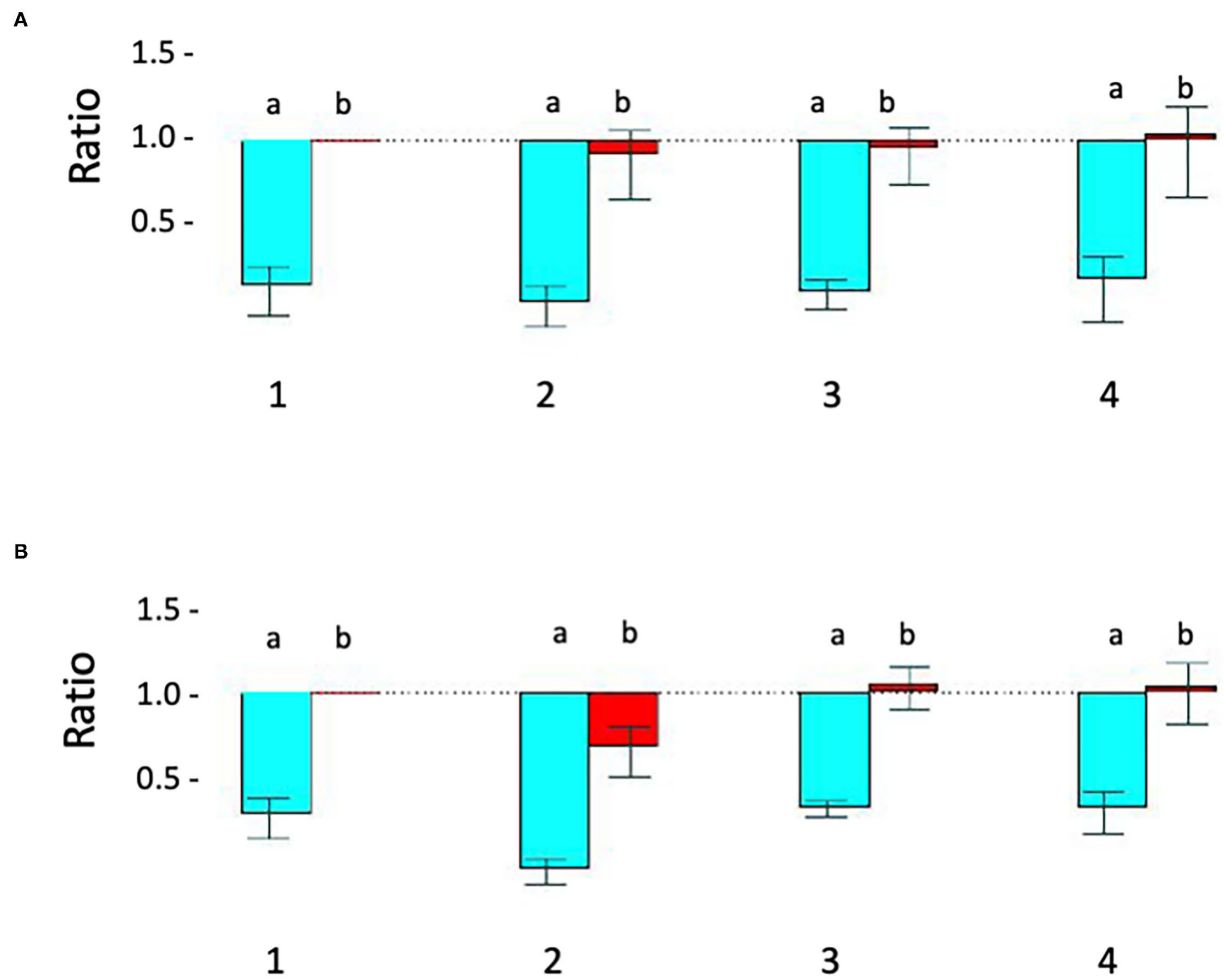
Relative quantitative –PCR [Q-PCR] confirms *STXBP1* deletion in proband 1 and demonstrates that it is *de novo*. **(A)** Q-PCR product using primer pair 1 (intron 1) did not detect the deletion in the proband 1 (#2), his parents (#3-mother, #4-father) and a pooled normal male sample control (#1). **(B)** With primer 2 (intron 19), Q-PCR product confirmed the *STXBP1* deletion in proband 1 (#2b) detected by microarray. No deletion was detected in the proband's mother (#3) and father (#4) or the control (#1). a = target/reference ratio (i.e., *STXBP1*/*FOXP2*); b = normalized ratio (i.e., = proband's a/normal controls a, mother's a/controls a and father's a/controls a). Error bars indicate average standard error of the mean obtained from normalization of triplicates for each sample. The test was reproducible and was repeated more than three times.

Proband 1: A 50 kb deletion including 56 oligonucleotide probes in chromosome region 9q34.11 was detected using CytoScan HD Array and ChAS analysis (Figure 1B). The genome coordinates of the deletion are 9q34.11(130 435 492–130 485 618)x1 [hg19]. This deletion resulted in loss of exons 13 through 20 and 3' downstream of the *STXBP1* gene (NM_003165, NM_001032221, OMIM# 602926). The Q-PCR studies confirmed the deletion in the proband and revealed the deletion was *de novo* (Figures 2A,B and Tables 1, 2). No identical deletion has been previously reported in literature or databases.

**Table 1:** Q-PCR analysis results for proband 1, his parents and the five pooled male controls using primer pair 1 at intron 1 of *STXBP1*.

**Sample**	**Reference gene**	**Target Cp-mean**	**Reference Cp-mean**	**Target/reference**	**Normalization**
Pooled male control	FOXP2	31.47	27.54	6.55E-02	1
Proband 1	FOXP2	31.64	27.46	5.54E-02	0.8458
Mother	FOXP2	31.73	27.22	6.43E-02	0.9817
Father	FOXP2	31.12	27.51	8.19E-02	1.2504

*The analyses showed that proband 1 and his parents have no deletion at intron 1 of the STXBP1 gene*.

**Table 2:** Q-PCR analysis results of proband 1, his parents and the five pooled male controls by using primer pair 2 at intron 19 of *STXBP1*.

**Sample**	**Reference gene**	**Target Cp- mean**	**Reference Cp-mean**	**Target/reference**	**Normalization**
Pooled male control	FOXP2	31.52	28.39	0.1138	1
Proband 1	FOXP2	32	27.46	4.32E-02	0.3796
Mother	FOXP2	30.73	27.77	0.1285	1.1292
Father	FOXP2	30.48	27.51	0.1276	1.1213

*The analysis confirmed the deletion detected by microarray analysis*.

Proband 2: Extracted DNA was sent to GeneDx, Inc (Gaithersburg, MD, USA) for testing with their infantile epilepsy 51-gene panel in 2013. A c.1663G>T (p.Glu555X) in exon 18 of *STXBP1* was detected. This variant is predicted to result in a premature stop codon at position 555 (nonsense mutation) in domain 2 (13). This variant has not been previously reported in the literature. Parental DNA sequencing studies revealed that this nonsense mutation was *de novo*.

### *STXBP1* Expression

**Figure 3:**
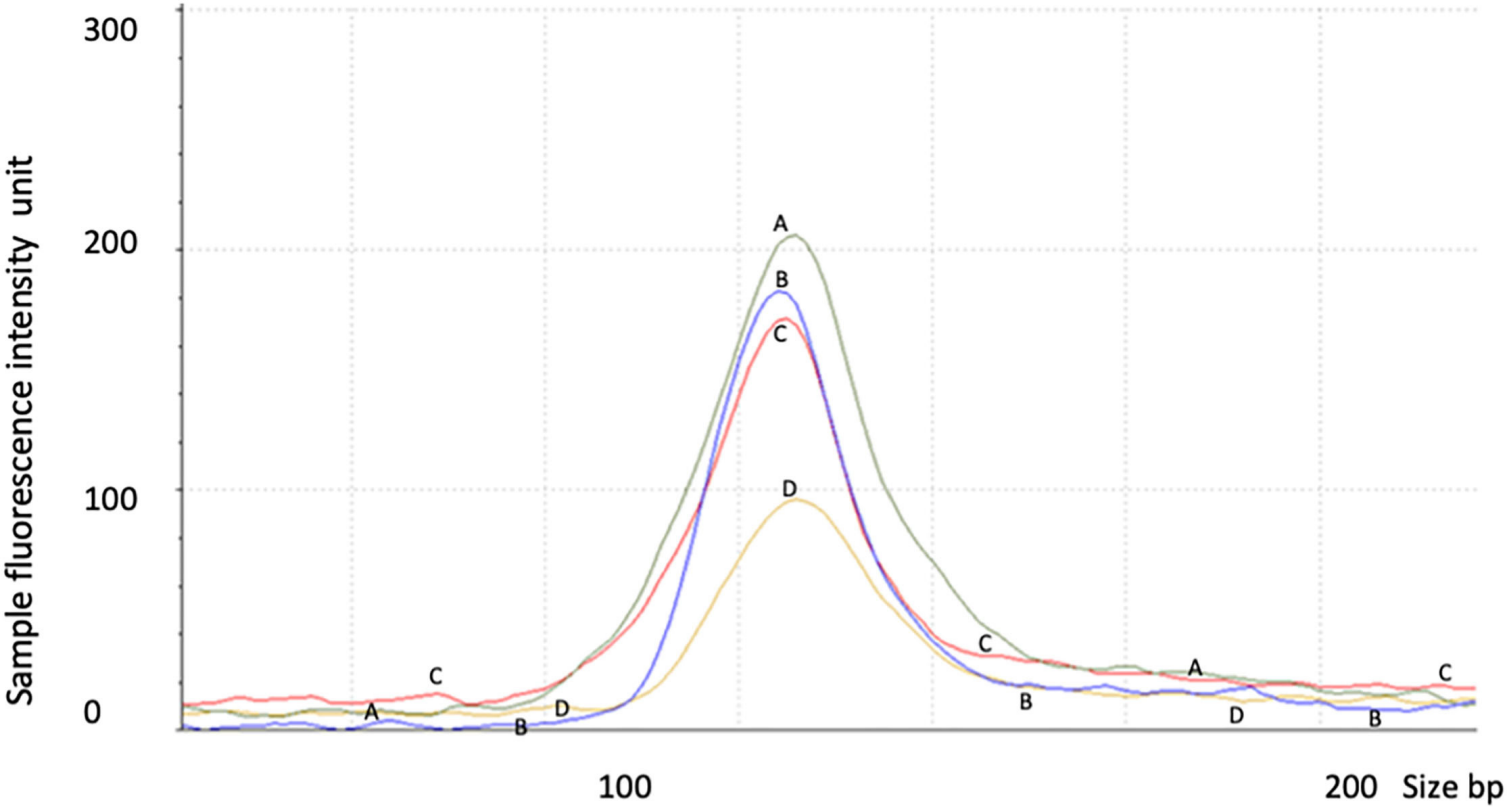
RNA decay observed in proband 1 with *STXBP1* deletion not observed in proband 2 with nonsense mutation. Control 1 (A) = 6 year old male, control 2 (C) = 1 year old male, proband 1 (D) = 2 year old male with partial deletion of *STXBP1*, and proband 2 (B) = a 6 year old male with c.1663G>T nonsense mutation of *STXBP1*. The data were averaged from triplicates for each sample. The test was repeated for at least three times.

Proband 1: RT-Q-PCR products from exons 5–6, outside of the deleted genomic region, showed that the expression level of the *STXBP1* was reduced to ~half of the expression level of the similar age normal control 2. This indicated that the deletion of exons 13–20 mediated one allele of RNA decay (Figure 3 and Table 3).

**Table 3:** RT-Q-PCR analyses of RNA expression from probands 1 and 2 relative to age-matched controls using primer pair 3 located at exons 5 and 6 of *STXBP1*.

**Sample**	**Age**	**Area**	**Proband1/sample (%)**	**Proband 1/control average (%)**	**Proband 2/sample (%)**	**Proband 2/control average (%)**
Control 1	6	0.118	44.10		96.60	
Control 2	1	0.102	51.00		111.80	
Proband 1	2	0.052	100.00	47.60	219.20	
Proband 2	6	0.114	45.60		100.00	104.20

*Proband 1 had ~51% of STXBP1 expression compared to the similar age control 1 (Expression=[Area of proband 1] / [Area of control 2]), or 47.6% of expression compared to the average of both controls (Expression=[Area of proband 1] / [Area of control 1 + area of control 2]). Proband 2 had ~97% of STXBP1 expression compared to same age control 1, or ~104% expression compared to the average of the both controls. Thus, the analysis detected RNA decay in proband 1 but not in proband 2. The results were reproducible. The tests were repeated at least three times*.

Proband 2: The expression level of *STXBP1* was not reduced (Figure 3 and Table 3). It is predicted to result in a truncated STXBP1 protein product with stop codon at amino acid position 555 in domain 2.

## Discussion

STXBP1 protein plays an important role in regulating the release of neurotransmitters during synaptic docking and fusion of the vesicle at the synaptic membrane. Mutations of *STXBP1* have been associated with autosomal dominant patterns associated with diverse neurodevelopmental phenotypes that include autism, intellectual disability, developmental delay and early infantile epileptic encephalopathy (7, 14). Approximately 136 point mutations (including splice, nonsense, missense), small indels (<100 kb, including insertions, deletions and duplications) and 26 cases with large copy number variants (CNVs) affecting *STXBP1* have been reported in the literature (14). Estimated incidence rate is 3.3–3.8 per 100,000 births (14). The mutations were distributed throughout all domains (domains 1 through 3) of the STXBP1 protein, and either reduced the amount of functional protein (haploinsufficiency) or caused an abnormal structural protein. An abnormal amount or structure of STXBP1 protein impairs the release of neurotransmitters that lead to uncontrolled excitation of neurons and seizures. However, the cause of developmental delay, intellectual disability and other phenotypes by altered STXBP1 protein is unknown. No established genotype and phenotype correlation has been established (15).

Our RT-Q-PCR assay demonstrated that the nonsense mutation detected at amino acid position 555 of STXBP1 in proband 2 did not lead to RNA decay. This mutation is located in exon 18, the second to last exon and ~40 nucleotides upstream of the last exon (exon 20) of *STXBP1* in *isoform b* and is consistent with the mechanism of escape from nonsense-mediated RNA decay where it is <50 nucleotides upstream of the last exon-exon junction (19). For *isoform a*, the mutation is located at the third last exon and approximately 37 nucleotides upstream of the second last exon (exon 19). However, the exon 20 in *isoform a* is not a coding exon, thus exon 19 is the last coding exon. It indicates that escape from nonsense-mediated RNA decay is possible where the nonsense mutation resides <50 nucleotides upstream of the last coding exon-exon junction even in the presence of additional non-coding exons at the 3′ downstream end of the coding exon. The escape of nonsense-mediated RNA decay predicts that truncated STXBP1 proteins could be produced. The predicted truncated proteins are short of 39 and 48 amino acids at the C-terminal end in domain 2 for *isoforms b and a*, respectively. This indicates that domain 2 is critical for the normal function of STXBP1. It has been proposed that missense mutations result in STXBP1 protein destabilization and are prone to misfolding, aggregation and degradation (20). Based on missense mutations that can cause symptoms as severe as frameshift and heterozygous deletion mutations, it was proposed that missense mutants likely act to deplete the normal SYXBP1 protein in a dominant negative way (14). Similarly, the truncated STXBP1 protein predicted in proband 2 may work in a dominant negative way that could lead to normal protein destabilization or inappropriate binding with syntaxin-1 protein or other SNARE (soluble N-ehtylmaleimide-sensitive factor attachment receptor) proteins required in synaptic vesicle docking, priming and fusion (20). Alternatively, the truncated STXBP1 protein in proband 2 may lose its function completely and result in haploinsufficiency as discussed in a patient with truncated STXBP1 with atypical Rett/Rett-like phenotypes (11). More studies of structural and functional assays on the truncated STXBP1 protein will be needed to clarify whether dominant negative or haploinsufficiency is the causative mechanism for epileptic encephalopathies.

In proband 1, who had a deletion of exons 13 through 20, our RT-Q-PCR assay suggested mediated RNA decay, expected to result in haploinsufficiency of STXBP1 protein. This finding demonstrates the haploinsufficiency mechanism for STXBP1 related disorders directly from RNA extracted from patient blood samples.

Proband 1 and proband 2 have common clinical features of developmental delay and intellectual disability. However, proband 2 presented with infantile spasms which were not noted in proband 1. Whether the difference is due to the different nature of mutations in *STXBP1* (deletion vs. nonsense mutation) remains unclear. Several studies revealed no correlation between the type of mutations or position within the gene with respect to epilepsy, cognitive abilities and other phenotypes (7, 20). Thus, the differences may have contribution from each individual's genetic background. Epistasis is a possible explanation for this, which the phenotypes depend on the presence of one or more modifier genes (21).

## Conclusion

We believe our RT-Q-PCR assay represents the first *ex vivo* assay to demonstrate that both truncation and deletion of *STXBP1*, resulting in truncated STXBP1 protein and reduced protein product, cause STXBP1 related disorders. Domain 2 of STXBP1 protein is critical for the normal function. The findings support haploinsufficiency and truncation/structure anomaly (either acts as dominant negative or haploinsufficiency) as mechanisms for the disorders. Our RNA assay also indicates that escape from nonsense-mediated RNA decay is possible where the nonsense mutation resides <50 nucleotides upstream of the last coding exon-exon junction even in the presence of additional non-coding exons at the 3' downstream of the coding exon. STXBP1 RNA and protein is highly expressed in brain, pancreas, soft tissue and lowly expressed in white blood cells (https://www.genecards.org/cgi-bin/carddisp.pl?gene=STXBP1). Our RT-Q-PCR assay supported that *STXPB1* RNA is also expressed in peripheral blood, and this provides a much more accessible way for *ex vivo* study.

## Data Availability Statement

The datasets presented in this study can be found in online repositories. The names of the repository/repositories and accession number(s) can be found below: https://www.ncbi.nlm.nih.gov/clinvar/variation/932281/.

## Ethics Statement

Ethical review and approval was not required for the study on human participants in accordance with the local legislation and institutional requirements. Written informed consent to participate in this study was provided by the participants' legal guardian/next of kin.

## Author Contributions

PY contributed to conception, design of the study, and wrote the first draft of the manuscript. ANP wrote clinical sections of the manuscript. ANP, CP, and SL contributed clinical and treatment data and edited the manuscript. SG contributed clinical data. RB conducted the experiments and analyzed the data. JHK discussed the study and edited the manuscript. All authors contributed to manuscript, read, and approved the submitted version.

## Funding

Both patients were funded for the clinical testing through Ontario Ministry of Health and Long-Term Care, Canada.

## Conflict of Interest

The authors declare that the research was conducted in the absence of any commercial or financial relationships that could be construed as a potential conflict of interest.

## Publisher's Note

All claims expressed in this article are solely those of the authors and do not necessarily represent those of their affiliated organizations, or those of the publisher, the editors and the reviewers. Any product that may be evaluated in this article, or claim that may be made by its manufacturer, is not guaranteed or endorsed by the publisher.
